# Addressing the Complications of Ebola and Other Viral Hemorrhagic Fever Infections: Using Insights from Bacterial and Fungal Sepsis

**DOI:** 10.1371/journal.ppat.1005088

**Published:** 2015-10-01

**Authors:** Judith Hellman

**Affiliations:** Department of Anesthesia and Perioperative Care, Division of Critical Care Medicine Faculty, Biomedical Sciences and Immunology Programs, University of California, San Francisco, San Francisco, California, United States of America; The Fox Chase Cancer Center, UNITED STATES

## Introduction

Research on Ebola virus (EBOV) has focused on preventing and controlling the infection using vaccines and antiviral therapies. Given the long-term challenge of the current epidemic and the likelihood of future outbreaks of viral hemorrhagic fevers caused by the filoviruses, including EBOV and Marburg virus, efforts should also focus on developing therapies to reduce the deadly complications of infection with these viruses [1,2]. There are striking similarities in the syndromes caused by bacterial and fungal sepsis [3–14] and by EBOV [15–27] (Table 1). Sepsis, defined as the systemic inflammatory response to infection, causes a spectrum of pathology ranging from mild, basic physiologic and laboratory derangements to shock, multiple organ failure, and death [3,7]. While the term “sepsis” is generally used in the context of bacterial and fungal infections, all microorganisms, including viruses, can cause sepsis. This Opinion argues that the wealth of knowledge about bacterial and fungal sepsis (herein referred to as “classical sepsis”) should be used to inform the development of adjunctive therapies to improve the outcome of EBOV and other viral hemorrhagic fevers.

**Table 1:** Similarities between Severe and Fatal EBOV and Classical Sepsis.*

Parameter	Similarities	References
Systemic Inflammation	Increased levels of pro-inflammatory cytokines (e.g., interleukin 6 [IL-6]), chemokines (IL-8), and the anti-inflammatory cytokine IL-10	**Classical Sepsis:** [5,10,11]; **EBOV:** [17,19,20]
Immune Dysfunction	Increased susceptibility to secondary bacterial infections, lymphocyte apoptosis	**Classical Sepsis:** [4,14]; **EBOV:** [23,28]
Coagulopathy	Increased D-dimers, thrombomodulin, ferritin, disseminated intravascular coagulation, thrombocytopenia	**Classical Sepsis:** [9,12]; **EBOV:** [17,18,20]
Endothelial Dysfunction	Vascular leak with hypovolemia	**Classical Sepsis:** [13]; **EBOV:** [23–25,27]
Organ Dysfunction	Renal insufficiency, hepatic dysfunction, respiratory failure, neurologic dysfunction	**Classical Sepsis:** [6,8,11]; **EBOV:** [21–25]

* Classical sepsis is defined as bacterial and fungal sepsis

## Pathophysiology of Classical Sepsis and EBOV

In classical sepsis, activation of innate immune pathways via pattern recognition receptors, such as the toll-like receptors (TLRs) and nucleotide-binding oligomerization domain (NOD)-like receptors, initiates systemic inflammation [29–31]. Maladaptive responses in sepsis cause excessive inflammation, endothelial dysfunction, coagulopathy, vascular leak, shock, and organ failure [11–13]. Analogous to the “cytokine storm” of classical sepsis, EBOV also causes systemic inflammation, endothelial dysfunction, coagulopathy, vascular leak, shock, and organ failure [17–25]. Fatal EBOV is associated with high levels of pro-inflammatory cytokines, chemokines, the anti-inflammatory cytokine IL-10, and nitric oxide [17,19,20]. Similar to classical sepsis, EBOV also causes immune suppression and a predisposition to secondary bacterial infections [11,15,23]. This latter complication has prompted the administration of empiric antibiotics to patients with EBOV [24–26]. It is possible that classical sepsis therapies may be beneficial in EBOV, in part because of their impact on the complications of secondary bacterial sepsis.

The mechanisms underlying immune and endothelial cell dysfunction and organ failure in EBOV have yet to be unraveled. Infection of monocytes, macrophages, and dendritic cells leads to acute inflammation [16]. Early activation and subsequent massive apoptosis of T-lymphocytes is associated with fatal outcomes in EBOV [17,32]. The innate immune system has been implicated in the beneficial and harmful responses to EBOV [15,27,33,34]. The EBOV glycoprotein (GP) is a putative TLR4 agonist [27,35]. The shed surface GP of EBOV has been detected in the blood during infection; it activates macrophages and endothelial cells and induces endothelial cytotoxicity and permeability [27,36]. Finally, EBOV suppresses antiviral immunity by interfering with signaling via the innate immune receptor, RIG-I, and by interfering with type I interferon (IFN) production and signaling [28,37–40]. The resultant increased viral load may further exacerbate inflammation by activating innate immune pathways and by causing cytolysis.

## Defining Approaches to the Viral Hemorrhagic Fevers Based on Classical Sepsis Research

**Fig 1:**
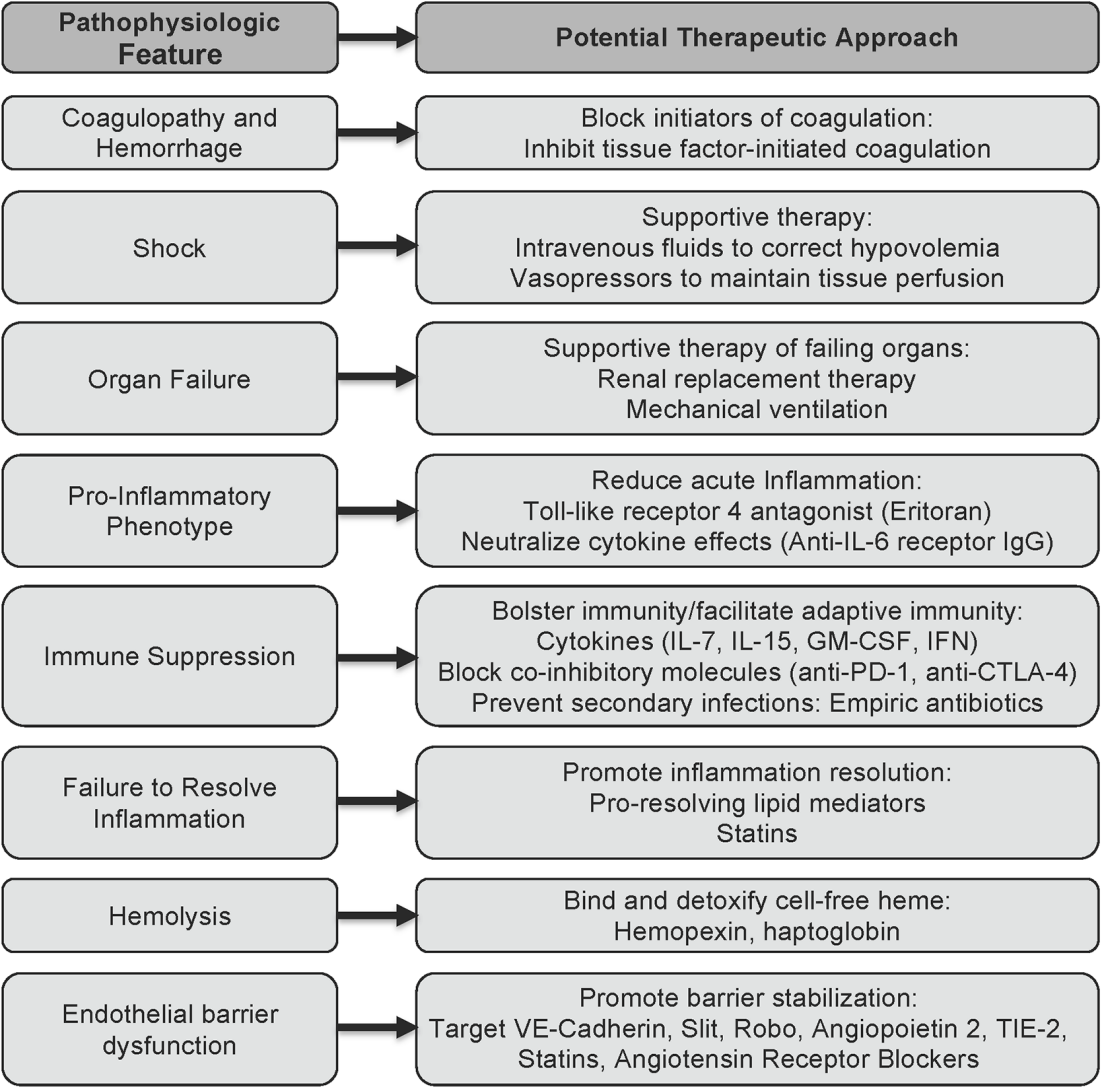
Potential approaches to sepsis caused by viral hemorrhagic fevers based on insights from classical sepsis. The schematic outlines potential approaches to reducing the downstream complications of the viral hemorrhagic fevers, based on what is currently known about the pathophysiology of EBOV sepsis and the state of the art of classical sepsis research.

Described below are strategies that have been studied in classical sepsis and could be applicable to sepsis caused by EBOV and other viral hemorrhagic fevers (Fig 1). Recognizing that some of these strategies will not be feasible in resource-limited areas, it would nonetheless be reasonable to move forward with preclinical and clinical studies to further characterize the pathophysiology and develop approaches to reduce the complications of EBOV sepsis.

### Supportive therapies

The Surviving Sepsis Campaign guidelines provide detailed instructions for the care of patients with sepsis based on state-of-the-art knowledge and therapeutics [41]. Mainstays of management include antibiotics, procedures to remove infectious foci, and the administration of basic supportive therapies (including fluids and vasopressors) to maintain tissue perfusion. More aggressive therapies are used to support patients through sepsis-induced organ failure. For example, ventilator support and renal replacement therapy are used to manage respiratory or renal failure, respectively [41]. Recent reports suggest that early administration of fluids, electrolytes, and nutrition reduces shock and organ failure in EBOV [23–26]. Thus, strong efforts should continue to be made towards making these basic therapies widely available. Although intensive care therapies such as mechanical ventilation and renal replacement therapy may not be available in all areas, they should be utilized in patients being cared for in countries with adequate resources, since recent data strongly suggest that these therapies improve the outcome of severe EBOV [23,25].

### Reduce acute inflammation

Numerous sepsis trials have used agents to neutralize specific pro-inflammatory mediators or to block inflammatory receptors. These directions have not yet been successful in reducing the mortality of classical sepsis [42]. In contrast to the heterogeneity of classical sepsis, EBOV sepsis is caused by a single microbe whose pathogenesis follows a reasonably characteristic course. Therefore, it is conceivable that the appropriately timed administration of an agent to neutralize the effects of an inflammatory mediator could be beneficial in EBOV. In this regard, high levels of IL-6 have been reported to correlate with fatal EBOV [19], and a humanized antibody to the IL-6 receptor has been used in humans to safely treat rheumatoid arthritis [43]. However, this approach is highly speculative, as high IL-6 levels have not yet been proven to mediate fatal outcomes of EBOV. Furthermore, although high cytokine levels correlate with fatal EBOV, paradoxically, an early robust pro-inflammatory response is associated with better outcomes in EBOV infection [32]. This observation suggests that early administration of agents to neutralize pro-inflammatory mediators such as IL-6 could, in fact, worsen outcomes by interfering with antiviral immunity and/or by increasing susceptibility to secondary bacterial infections.

One intriguing possibility would be to use a TLR4 antagonist such as Eritoran to reduce activation of leukocytes and endothelial cells. A TLR4 antagonist might reduce systemic inflammation and endothelial dysfunction induced by the EBOV-shed GP, a putative TLR4 agonist [27,35], without interfering with the initiation of protective responses via other intracellular innate immune receptors, such as RIG-I. Despite the recent negative Phase III randomized controlled trial (RCT) in classical sepsis [44], and given the safety of Eritoran in humans, it would be reasonable to study this approach in preclinical studies and to consider a limited trial in humans with EBOV.

### Reverse immune suppression

Sepsis and EBOV disease cause immune suppression. Current sepsis studies are focused on restoring immune function using cytokines (e.g., IL-7, IL-15, GM-CSF, and type I IFN) or blocking co-inhibitory molecules (e.g., PD-1 and CTLA-4) [14]. Early treatment with immune-enhancing agents may promote earlier adaptive immunity and facilitate more rapid resolution of infection. This approach might be beneficial in EBOV, in which higher viral loads correlate with increased mortality [20,22].

### Promote inflammation resolution

The failure to resolve acute inflammation is believed to contribute to poor outcomes in sepsis. Specialized pro-resolving lipid mediators, including resolvins, maresins, and lipoxins, can reduce inflammation without compromising anti-microbial defenses and are being investigated in preclinical sepsis studies [45–50]. The endocannabinoids, another class of endogenous lipids, have received attention recently for their ability to modulate inflammation [51–53]. The availability and safety of plant-derived cannabinoids suggests that, if effective, they could be viable treatment options. Statins have inflammation-resolving properties and have been proposed for EBOV [45,54]. However, some recent meta-analyses of RCTs have failed to show a survival benefit for statins in classical sepsis [55,56].

### Corticosteroids

Following the Surviving Sepsis Campaign guidelines [41], corticosteroids could potentially be used in patients with EBOV and refractory shock. However, although low-dose corticosteroids can reverse shock, recent meta-analyses of RCTs in classical sepsis have failed to show that corticosteroids improve survival [57,58]. Based on the lack of definitive proof that corticosteroids improve outcomes in sepsis and the potential for corticosteroids to impair adaptive immunity and exacerbate gastrointestinal bleeding, their routine use in EBOV is not recommended.

### Modulate coagulation pathways

The coagulopathies of classical sepsis and EBOV are initiated through activation of tissue factor [9,59]. At the extreme, these syndromes cause disseminated intravascular coagulation (DIC). The mixed coagulopathy of EBOV presents a conundrum as to whether to target coagulation, anticoagulation, or fibrinolysis. Studies in EBOV have focused on modulating proximal coagulation pathways, which may reduce bleeding and microvascular thrombosis. Treatment with an inhibitor of tissue factor-initiated coagulation was reported to improve outcomes in nonhuman primates with EBOV, suggesting that this may be a viable approach in humans [60]. Neither activated protein C nor tissue factor pathway inhibitor (TFPI) seem appropriate for testing in EBOV based on negative RCTs in classical sepsis [61,62] and concerns that they may exacerbate bleeding.

### Stabilize the endothelium

The proteins VE-cadherin, Slit, Robo, Angiopoietin 2, and TIE2—all involved in maintaining the endothelial barrier—are being explored as therapeutic targets in classical sepsis [63–65] and are potential targets in EBOV. Combined treatment with statins and angiotensin receptor blockers, which each have endothelial stabilizing effects, has also been proposed to treat the vascular leak associated with EBOV [54].

### Bind cell-free heme

The syndrome of DIC causes hemolysis with hemoglobin release. Cell-free heme potentiates inflammation induced by microbial products [66,67]. Recent reports that the heme binding proteins hemopexin and haptoglobin are protective in sepsis models [66–68] suggest that these proteins could be viable adjuvant therapies for EBOV.

## Concluding Remarks

Although we are encouraged by the reduction in the current EBOV epidemic, recently, cases of EBOV have been reported again in Liberia [69], and it is likely that there will be future outbreaks of EBOV and other viral hemorrhagic fevers. Numerous lives may again be lost while developing a vaccine. Insights from classical sepsis research could be used to develop approaches to address the complications of the sepsis that can be common to the viral hemorrhagic fevers. These approaches could be implemented well before a vaccine is available and could hugely impact the morbidity and mortality of EBOV and other viral hemorrhagic fevers.

## References

[1] Team WHOER (2014) Ebola virus disease in West Africa—the first 9 months of the epidemic and forward projections. N Engl J Med 371: 1481–1495. 10.1056/NEJMoa1411100 25244186 PMC4235004

[2] Team WHOER, Agua-Agum J, Ariyarajah A, Aylward B, Blake IM, et al (2015) West African Ebola epidemic after one year—slowing but not yet under control. N Engl J Med 372: 584–587. 10.1056/NEJMc1414992 25539446 PMC4368109

[3] Bone RC, Balk RA, Cerra FB, Dellinger RP, Fein AM, et al (1992) Definitions for sepsis and organ failure and guidelines for the use of innovative therapies in sepsis. The ACCP/SCCM Consensus Conference Committee. American College of Chest Physicians/Society of Critical Care Medicine. Chest 101: 1644–1655. 1303622 10.1378/chest.101.6.1644

[4] Hotchkiss RS, Swanson PE, Freeman BD, Tinsley KW, Cobb JP, et al (1999) Apoptotic cell death in patients with sepsis, shock, and multiple organ dysfunction. Crit Care Med 27: 1230–1251. 10446814 10.1097/00003246-199907000-00002

[5] Gogos CA, Drosou E, Bassaris HP, Skoutelis A (2000) Pro- versus anti-inflammatory cytokine profile in patients with severe sepsis: a marker for prognosis and future therapeutic options. J Infect Dis 181: 176–180. 10608764 10.1086/315214

[6] Angus DC, Linde-Zwirble WT, Lidicker J, Clermont G, Carcillo J, et al (2001) Epidemiology of severe sepsis in the United States: analysis of incidence, outcome, and associated costs of care. Crit Care Med 29: 1303–1310. 11445675 10.1097/00003246-200107000-00002

[7] Levy MM, Fink MP, Marshall JC, Abraham E, Angus D, et al (2003) 2001 SCCM/ESICM/ACCP/ATS/SIS International Sepsis Definitions Conference. Crit Care Med 31: 1250–1256. 12682500 10.1097/01.CCM.0000050454.01978.3B

[8] Martin GS, Mannino DM, Eaton S, Moss M (2003) The epidemiology of sepsis in the United States from 1979 through 2000. N Engl J Med 348: 1546–1554. 12700374 10.1056/NEJMoa022139

[9] Levi M, de Jonge E, van der Poll T (2003) Sepsis and disseminated intravascular coagulation. J Thromb Thrombolysis 16: 43–47. 14760211 10.1023/B:THRO.0000014592.27892.11

[10] Kellum JA, Kong L, Fink MP, Weissfeld LA, Yealy DM, et al (2007) Understanding the inflammatory cytokine response in pneumonia and sepsis: results of the Genetic and Inflammatory Markers of Sepsis (GenIMS) Study. Arch Intern Med 167: 1655–1663. 17698689 10.1001/archinte.167.15.1655 PMC4495652

[11] Rittirsch D, Flierl MA, Ward PA (2008) Harmful molecular mechanisms in sepsis. Nat Rev Immunol 8: 776–787. 10.1038/nri2402 18802444 PMC2786961

[12] Schouten M, Wiersinga WJ, Levi M, van der Poll T (2008) Inflammation, endothelium, and coagulation in sepsis. J Leukoc Biol 83: 536–545. 18032692 10.1189/jlb.0607373

[13] Kumar P, Shen Q, Pivetti CD, Lee ES, Wu MH, et al (2009) Molecular mechanisms of endothelial hyperpermeability: implications in inflammation. Expert Rev Mol Med 11: e19 10.1017/S1462399409001112 19563700 PMC2828491

[14] Hutchins NA, Unsinger J, Hotchkiss RS, Ayala A (2014) The new normal: immunomodulatory agents against sepsis immune suppression. Trends Mol Med 20: 224–233. 10.1016/j.molmed.2014.01.002 24485901 PMC3976785

[15] Mahanty S, Bray M (2004) Pathogenesis of filoviral haemorrhagic fevers. Lancet Infect Dis 4: 487–498. 15288821 10.1016/S1473-3099(04)01103-X

[16] Feldmann H, Geisbert TW (2011) Ebola haemorrhagic fever. Lancet 377: 849–862. 10.1016/S0140-6736(10)60667-8 21084112 PMC3406178

[17] Sanchez A, Lukwiya M, Bausch D, Mahanty S, Sanchez AJ, et al (2004) Analysis of human peripheral blood samples from fatal and nonfatal cases of Ebola (Sudan) hemorrhagic fever: cellular responses, virus load, and nitric oxide levels. J Virol 78: 10370–10377. 15367603 10.1128/JVI.78.19.10370-10377.2004 PMC516433

[18] Rollin PE, Bausch DG, Sanchez A (2007) Blood chemistry measurements and D-Dimer levels associated with fatal and nonfatal outcomes in humans infected with Sudan Ebola virus. J Infect Dis 196 Suppl 2: S364–371. 17940972 10.1086/520613

[19] Hutchinson KL, Rollin PE (2007) Cytokine and chemokine expression in humans infected with Sudan Ebola virus. J Infect Dis 196 Suppl 2: S357–363. 17940971 10.1086/520611

[20] McElroy AK, Erickson BR, Flietstra TD, Rollin PE, Nichol ST, et al (2014) Ebola hemorrhagic Fever: novel biomarker correlates of clinical outcome. J Infect Dis 210: 558–566. 10.1093/infdis/jiu088 24526742 PMC4172044

[21] Chertow DS, Kleine C, Edwards JK, Scaini R, Giuliani R, et al (2014) Ebola virus disease in West Africa—clinical manifestations and management. N Engl J Med 371: 2054–2057. 10.1056/NEJMp1413084 25372854

[22] Schieffelin JS, Shaffer JG, Goba A, Gbakie M, Gire SK, et al (2014) Clinical illness and outcomes in patients with Ebola in Sierra Leone. N Engl J Med 371: 2092–2100. 10.1056/NEJMoa1411680 25353969 PMC4318555

[23] Kreuels B, Wichmann D, Emmerich P, Schmidt-Chanasit J, de Heer G, et al (2014) A case of severe Ebola virus infection complicated by gram-negative septicemia. N Engl J Med 371: 2394–2401. 10.1056/NEJMoa1411677 25337633

[24] Lyon GM, Mehta AK, Varkey JB, Brantly K, Plyler L, et al (2014) Clinical care of two patients with Ebola virus disease in the United States. N Engl J Med 371: 2402–2409. 10.1056/NEJMoa1409838 25390460

[25] Wolf T, Kann G, Becker S, Stephan C, Brodt HR, et al (2015) Severe Ebola virus disease with vascular leakage and multiorgan failure: treatment of a patient in intensive care. Lancet 385: 1428–1435. 10.1016/S0140-6736(14)62384-9 25534190

[26] Ansumana R, Jacobsen KH, Sahr F, Idris M, Bangura H, et al (2015) Ebola in Freetown area, Sierra Leone—a case study of 581 patients. N Engl J Med 372: 587–588. 10.1056/NEJMc1413685 25539447

[27] Escudero-Perez B, Volchkova VA, Dolnik O, Lawrence P, Volchkov VE (2014) Shed GP of Ebola virus triggers immune activation and increased vascular permeability. PLoS Pathog 10: e1004509 10.1371/journal.ppat.1004509 25412102 PMC4239094

[28] Mahanty S, Hutchinson K, Agarwal S, McRae M, Rollin PE, et al (2003) Cutting edge: impairment of dendritic cells and adaptive immunity by Ebola and Lassa viruses. J Immunol 170: 2797–2801. 12626527 10.4049/jimmunol.170.6.2797

[29] Hotchkiss RS, Karl IE (2003) The pathophysiology and treatment of sepsis. N Engl J Med 348: 138–150. 12519925 10.1056/NEJMra021333

[30] Beutler BA (2009) TLRs and innate immunity. Blood 113: 1399–1407. 10.1182/blood-2008-07-019307 18757776 PMC2644070

[31] Opitz B, Eitel J, Meixenberger K, Suttorp N (2009) Role of Toll-like receptors, NOD-like receptors and RIG-I-like receptors in endothelial cells and systemic infections. Thromb Haemost 102: 1103–1109. 10.1160/TH09-05-0323 19967140

[32] Baize S, Leroy EM, Georges AJ, Georges-Courbot MC, Capron M, et al (2002) Inflammatory responses in Ebola virus-infected patients. Clin Exp Immunol 128: 163–168. 11982604 10.1046/j.1365-2249.2002.01800.x PMC1906357

[33] Gupta M, Mahanty S, Ahmed R, Rollin PE (2001) Monocyte-derived human macrophages and peripheral blood mononuclear cells infected with ebola virus secrete MIP-1alpha and TNF-alpha and inhibit poly-IC-induced IFN-alpha in vitro. Virology 284: 20–25. 11352664 10.1006/viro.2001.0836

[34] Hensley LE, Young HA, Jahrling PB, Geisbert TW (2002) Proinflammatory response during Ebola virus infection of primate models: possible involvement of the tumor necrosis factor receptor superfamily. Immunol Lett 80: 169–179. 11803049 10.1016/s0165-2478(01)00327-3

[35] Dolnik O, Volchkova V, Garten W, Carbonnelle C, Becker S, et al (2004) Ectodomain shedding of the glycoprotein GP of Ebola virus. EMBO J 23: 2175–2184. 15103332 10.1038/sj.emboj.7600219 PMC424403

[36] Yang ZY, Duckers HJ, Sullivan NJ, Sanchez A, Nabel EG, et al (2000) Identification of the Ebola virus glycoprotein as the main viral determinant of vascular cell cytotoxicity and injury. Nat Med 6: 886–889. 10932225 10.1038/78645

[37] Basler CF, Mikulasova A, Martinez-Sobrido L, Paragas J, Muhlberger E, et al (2003) The Ebola virus VP35 protein inhibits activation of interferon regulatory factor 3. J Virol 77: 7945–7956. 12829834 10.1128/JVI.77.14.7945-7956.2003 PMC161945

[38] Bosio CM, Aman MJ, Grogan C, Hogan R, Ruthel G, et al (2003) Ebola and Marburg viruses replicate in monocyte-derived dendritic cells without inducing the production of cytokines and full maturation. J Infect Dis 188: 1630–1638. 14639532 10.1086/379199

[39] Kash JC, Muhlberger E, Carter V, Grosch M, Perwitasari O, et al (2006) Global suppression of the host antiviral response by Ebola- and Marburgviruses: increased antagonism of the type I interferon response is associated with enhanced virulence. J Virol 80: 3009–3020. 16501110 10.1128/JVI.80.6.3009-3020.2006 PMC1395418

[40] Cardenas WB, Loo YM, Gale M Jr., Hartman AL, Kimberlin CR, et al (2006) Ebola virus VP35 protein binds double-stranded RNA and inhibits alpha/beta interferon production induced by RIG-I signaling. J Virol 80: 5168–5178. 16698997 10.1128/JVI.02199-05 PMC1472134

[41] Dellinger RP, Levy MM, Rhodes A, Annane D, Gerlach H, et al (2013) Surviving sepsis campaign: international guidelines for management of severe sepsis and septic shock: 2012. Crit Care Med 41: 580–637. 10.1097/CCM.0b013e31827e83af 23353941

[42] Angus DC (2011) The search for effective therapy for sepsis: back to the drawing board? JAMA 306: 2614–2615. 10.1001/jama.2011.1853 22187284

[43] Nishimoto N, Miyasaka N, Yamamoto K, Kawai S, Takeuchi T, et al (2009) Study of active controlled tocilizumab monotherapy for rheumatoid arthritis patients with an inadequate response to methotrexate (SATORI): significant reduction in disease activity and serum vascular endothelial growth factor by IL-6 receptor inhibition therapy. Mod Rheumatol 19: 12–19. 10.1007/s10165-008-0125-1 18979150 PMC2638601

[44] Opal SM, Laterre PF, Francois B, LaRosa SP, Angus DC, et al (2013) Effect of eritoran, an antagonist of MD2-TLR4, on mortality in patients with severe sepsis: the ACCESS randomized trial. JAMA 309: 1154–1162. 10.1001/jama.2013.2194 23512062

[45] Spite M, Serhan CN (2010) Novel lipid mediators promote resolution of acute inflammation: impact of aspirin and statins. Circ Res 107: 1170–1184. 10.1161/CIRCRESAHA.110.223883 21071715 PMC3027152

[46] Walker J, Dichter E, Lacorte G, Kerner D, Spur B, et al (2011) Lipoxin a4 increases survival by decreasing systemic inflammation and bacterial load in sepsis. Shock 36: 410–416. 10.1097/SHK.0b013e31822798c1 21701419

[47] Li Y, Dalli J, Chiang N, Baron RM, Quintana C, et al (2013) Plasticity of leukocytic exudates in resolving acute inflammation is regulated by MicroRNA and proresolving mediators. Immunity 39: 885–898. 10.1016/j.immuni.2013.10.011 24238341 PMC3888517

[48] Chatterjee A, Sharma A, Chen M, Toy R, Mottola G, et al (2014) The Pro-Resolving Lipid Mediator Maresin 1 (MaR1) Attenuates Inflammatory Signaling Pathways in Vascular Smooth Muscle and Endothelial Cells. PLoS One 9: e113480 10.1371/journal.pone.0113480 25409514 PMC4237455

[49] Fullerton JN, O'Brien AJ, Gilroy DW (2014) Lipid mediators in immune dysfunction after severe inflammation. Trends Immunol 35: 12–21. 10.1016/j.it.2013.10.008 24268519 PMC3884129

[50] Buckley CD, Gilroy DW, Serhan CN (2014) Proresolving lipid mediators and mechanisms in the resolution of acute inflammation. Immunity 40: 315–327. 10.1016/j.immuni.2014.02.009 24656045 PMC4004957

[51] Nagarkatti P, Pandey R, Rieder SA, Hegde VL, Nagarkatti M (2009) Cannabinoids as novel anti-inflammatory drugs. Future Med Chem 1: 1333–1349. 10.4155/fmc.09.93 20191092 PMC2828614

[52] Wilhelmsen K, Khakpour S, Tran A, Sheehan K, Schumacher M, et al (2014) The endocannabinoid/endovanilloid N-arachidonoyl dopamine (NADA) and synthetic cannabinoid WIN55,212–2 abate the inflammatory activation of human endothelial cells. J Biol Chem 289: 13079–13100. 10.1074/jbc.M113.536953 24644287 PMC4036321

[53] Tschop J, Kasten KR, Nogueiras R, Goetzman HS, Cave CM, et al (2009) The cannabinoid receptor 2 is critical for the host response to sepsis. J Immunol 183: 499–505. 10.4049/jimmunol.0900203 19525393 PMC2763235

[54] Fedson DS, Jacobson JR, Rordam OM, Opal SM (2015) Treating the Host Response to Ebola Virus Disease with Generic Statins and Angiotensin Receptor Blockers. MBio 6(3):e00716 10.1128/mBio.00716-15 26106080 PMC4479704

[55] Thomas G, Hraiech S, Loundou A, Truwit J, Kruger P, et al (2015) Statin therapy in critically-ill patients with severe sepsis: a review and meta-analysis of randomized clinical trials. Minerva Anestesiol 81(8):921–30. 25690048

[56] Deshpande A, Pasupuleti V, Rothberg MB (2015) Statin therapy and mortality from sepsis: a meta-analysis of randomized trials. Am J Med 128: 410–417 e411. 10.1016/j.amjmed.2014.10.057 25526798

[57] Annane D, Sebille V, Charpentier C, Bollaert PE, Francois B, et al (2002) Effect of treatment with low doses of hydrocortisone and fludrocortisone on mortality in patients with septic shock. JAMA 288: 862–871. 12186604 10.1001/jama.288.7.862

[58] Sprung CL, Annane D, Keh D, Moreno R, Singer M, et al (2008) Hydrocortisone therapy for patients with septic shock. N Engl J Med 358: 111–124. 10.1056/NEJMoa071366 18184957

[59] Geisbert TW, Young HA, Jahrling PB, Davis KJ, Kagan E, et al (2003) Mechanisms underlying coagulation abnormalities in ebola hemorrhagic fever: overexpression of tissue factor in primate monocytes/macrophages is a key event. J Infect Dis 188: 1618–1629. 14639531 10.1086/379724

[60] Geisbert TW, Hensley LE, Jahrling PB, Larsen T, Geisbert JB, et al (2003) Treatment of Ebola virus infection with a recombinant inhibitor of factor VIIa/tissue factor: a study in rhesus monkeys. Lancet 362: 1953–1958. 14683653 10.1016/S0140-6736(03)15012-X

[61] Ranieri VM, Thompson BT, Barie PS, Dhainaut JF, Douglas IS, et al (2012) Drotrecogin alfa (activated) in adults with septic shock. N Engl J Med 366: 2055–2064. 10.1056/NEJMoa1202290 22616830

[62] Abraham E, Reinhart K, Opal S, Demeyer I, Doig C, et al (2003) Efficacy and safety of tifacogin (recombinant tissue factor pathway inhibitor) in severe sepsis: a randomized controlled trial. JAMA 290: 238–247. 12851279 10.1001/jama.290.2.238

[63] Dejana E, Orsenigo F, Lampugnani MG (2008) The role of adherens junctions and VE-cadherin in the control of vascular permeability. J Cell Sci 121: 2115–2122. 10.1242/jcs.017897 18565824

[64] London NR, Zhu W, Bozza FA, Smith MC, Greif DM, et al (2010) Targeting Robo4-dependent Slit signaling to survive the cytokine storm in sepsis and influenza. Sci Transl Med 2: 23ra19 10.1126/scitranslmed.3000678 20375003 PMC2875996

[65] Ziegler T, Horstkotte J, Schwab C, Pfetsch V, Weinmann K, et al (2013) Angiopoietin 2 mediates microvascular and hemodynamic alterations in sepsis. J Clin Invest 123(8):3436–3445. 10.1172/JCI66549 PMC3726157 23863629

[66] Larsen R, Gozzelino R, Jeney V, Tokaji L, Bozza FA, et al (2010) A central role for free heme in the pathogenesis of severe sepsis. Sci Transl Med 2: 51ra71 10.1126/scitranslmed.3001118 20881280

[67] Lin T, Sammy F, Yang H, Thundivalappil S, Hellman J, et al (2012) Identification of hemopexin as an anti-inflammatory factor that inhibits synergy of hemoglobin with HMGB1 in sterile and infectious inflammation. J Immunol 189: 2017–2022. 10.4049/jimmunol.1103623 22772444 PMC3426910

[68] Arredouani MS, Kasran A, Vanoirbeek JA, Berger FG, Baumann H, et al (2005) Haptoglobin dampens endotoxin-induced inflammatory effects both in vitro and in vivo. Immunology 114: 263–271. 15667571 10.1111/j.1365-2567.2004.02071.x PMC1782073

[69] Gulland A (2015) Liberia confirms Ebola case two months after being declared free of the disease. BMJ 350: h3620 10.1136/bmj.h3620 26138714

